# Is Navigated Transcranial Magnetic Stimulation Capable of Detecting Different Motor Cell Clusters Within the Precentral Gyrus Using a Single Pulse Protocol?

**DOI:** 10.1002/brb3.71313

**Published:** 2026-03-12

**Authors:** Gregor Durner, Georg Colbus, Vera Marschal, Giulia Sestito, Hans‐Georg Kesseler, Lennart Sannwald, Nadja Grübel, Christian Rainer Wirtz, Andrej Pala, Ralf Becker

**Affiliations:** ^1^ Department of Neurosurgery University of Ulm Günzburg Germany; ^2^ Department of Neurosurgery University of Augsburg Augsburg Germany

**Keywords:** nTMS, M1, hand knob, motor mapping

## Abstract

**Objective:**

In 2021, Rossi et al. developed a cortical mapping algorithm that enabled a reliable mapping of an anatomical subdivision of the precentral gyrus. This approach allowed for the differentiation between an anterior subregion, characterized by lower excitability and slower motor responses, and a posterior subregion (named “M1 proper”), exhibiting higher excitability and faster motor responses. Based on these findings, we conducted a navigated transcranial magnetic stimulation (nTMS) study to investigate whether this anatomical subdivision can also be identified using a non‐invasive mapping technique.

**Methods:**

Fifteen healthy volunteers were examined. Using nTMS, the motor cortex at the hand knob area was subdivided into four distinct strips aligned parallel to the central sulcus: s, precentral non‐proper, precentral proper, and postcentral. After determining the resting motor threshold (RMT), 10 stimulation points in each strip were stimulated with 150% RMT. Electromyographic (EMG) amplitudes and latencies were recorded and compared across the four stimulation strips to detect potential differences.

**Results:**

Although descriptively higher mean EMG amplitudes were observed in the precentral proper strip, generalized linear mixed‐effects modeling revealed no statistically significant differences in EMG amplitude between the precentral proper and non‐proper regions, nor across any of the four stimulation strips.

**Conclusions:**

Using the applied protocol, a clear delineation between a functionally separated ventral and dorsal subdivision of the precentral gyrus could not be observed. However, it is noteworthy that the application of relatively high stimulation intensity may have masked subtle differences. Further examinations at the lower end of the stimulation spectrum (e.g., 90% or 100% RMT) or a specific paired pulse protocol may allow for a more precise differentiation between these two anatomical areas.

## Abstract 2

Using navigated TMS, we investigated whether functional subdivisions of the primary motor cortex can be identified non‐invasively. No significant differences in excitability or latency were found between precentral subregions, suggesting that standard stimulation protocols may not reliably differentiate M1 proper from adjacent areas.

## 1 Introduction

Our present understanding of motor neuroanatomy is primarily founded on the pioneering work of Penfield and Boldrey in 1937 (Penfield and Boldrey 1937). Subsequent advancements in our comprehension of motor hodotopy have led to the development of increasingly sophisticated mapping techniques, applicable in both conscious and asleep subjects (Ghimire et al. 2021; Rossi et al. 2022; Suarez‐Meade et al. 2020; Szelényi et al. 2010). In 2021, Rossi et al. (2021) successfully translated findings from animal studies on the anatomical subdivision of the primary motor cortex into neurosurgical practice . This work built upon previous research in non‐human primates by Witham, Rathelot, and colleagues, who demonstrated that the primary motor cortex (M1) can be divided into an anterior region (named “M1 non‐proper” in Rossi's nomenclature), characterized by lower excitability and slower motor responses, and a posterior region (“M1 proper”), which exhibits higher excitability and faster motor responses (Rathelot and Strick 2009; Rossi et al. 2021; Witham et al. 2016). Based on these insights, they developed a stimulation algorithm to reliably distinguish these subdivisions during neurosurgical procedures, and thus allowing for safer glioma resection.

Similarly, since the first studies on non‐invasive motor mapping of the hand area, transcranial magnetic stimulation (TMS) mapping has undergone continuous refinement (Barker et al. 1985; Di Lazzaro et al. 2004). Specifically, stimulation parameters, such as current direction, pulse waveform, and stimulator types, have been optimized to enhance the precision and efficacy of the stimulation process (Bashir et al. 2013; Kammer et al. 2001; Souza et al. 2018).

In this study, we designed a navigated TMS (nTMS) protocol to investigate whether the anatomical subdivision of the precentral gyrus—previously identified through invasive methods—can be reliably reproduced using a non‐invasive mapping approach. If such a differentiation could be achieved using a simple single‐pulse protocol, this would constitute a valuable advancement for clinical preoperative motor mapping in patients undergoing planned glioma resections within this region.

## 2 Methods

### 2.1 Patients

Fifteen healthy subjects between 23 and 30 years old (seven men and eight women) were included in this examination after giving informed, written consent. The study was approved by our local ethics committee (Ulm University, Nr. 333/24). Since the aim of the study was to demonstrate a presumed anatomical normative structure, preferably young and healthy subjects were selected.

### 2.2 Stimulation Protocol

Motor mapping was conducted using the Nexstim NBS 5.0 system (Nexstim Oy, Helsinki, Finland), equipped with a figure‐eight coil. The maximum electric field strength is specified as 172 V/m by the manufacturer, with a maximum magnetic field strength of 1.42 T. The system delivers biphasic pulses with a pulse duration of 230 µs. Electromyography (EMG) recordings were obtained using surface electrodes (NeuroTab, Spes Medica, Genova, Italy).

EMG responses were automatically classified as positive if they exceeded a threshold of 50 µV, as determined by the device's built‐in stimulation software. However, all motor evoked potentials (MEPs) were retrospectively re‐evaluated, and responses below the 50 µV threshold were also classified as positive if a reliable EMG response could be identified. The study was conducted as a single‐session examination per subject, with mapping restricted to the dominant hemisphere.

The following describes the mapping routine used to identify the hot spot:

The functional hand motor area was first localized based on the characteristic omega‐shaped knob within the precentral gyrus, which serves as an anatomical landmark for the hand representation area. The search for the hotspot of the abductor digit minimi (ADM) muscle was initiated within this region. Alternatively abductor pollicis brevis (APB) muscle was used.

The stimulator output was initially adjusted to generate an estimated electric field strength of 80–100 V/m at the cortical surface, corresponding to roughly 35%–40% of the maximum stimulator output (MSO). A coarse mapping was then performed by applying single pulses across the precentral gyrus, moving medially and laterally along the central sulcus (CS) while maintaining a coil orientation perpendicular to the CS. The intensity was kept constant, and sites that evoked the highest MEPs were noted. The stimulation area was extended until no MEPs could be elicited. The location that produced the largest and most consistent ADM MEPs was defined as the ADM hotspot.

Resting motor threshold (RMT) was then determined in the ADM hotspot area using a standardized threshold‐hunting algorithm provided by the manufacturer.

**FIGURE 1:**
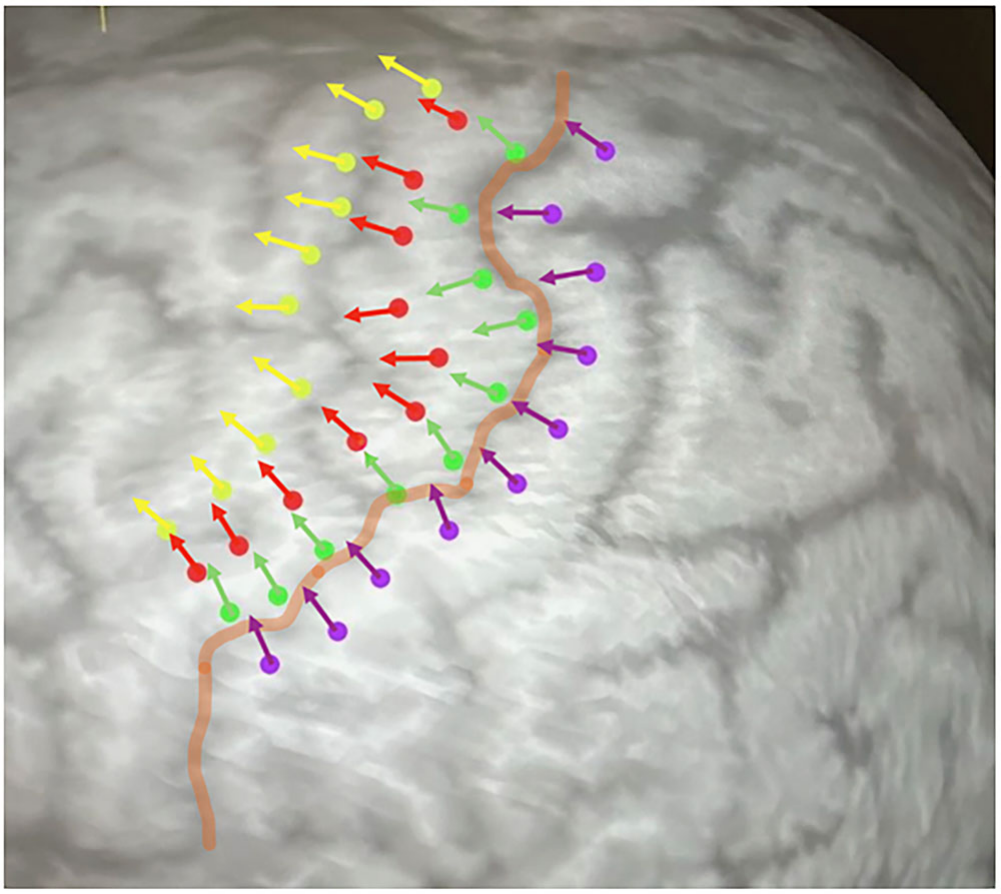
For detailed motor mapping, the hand knob region was subdivided into four anatomical strips parallel to the central sulcus (orange line): anteprecentral (yellow), precentral M1 non‐proper (red), precentral M1 proper (green), and postcentral (purple). Ten stimulation sites per strip were sequentially stimulated at 150% RMT, starting from the basal (temporal) position, beginning with M1 non‐proper, followed by M1 proper, then the anteprecentral, and finally the postcentral line, with electromyographic (EMG) responses recorded for offline analysis of amplitudes and latencies.

Subsequently, motor mapping of the hand knob area was performed following a standardized protocol (see Figure 1). The motor cortex at the hand knob level was systematically divided into four distinct anatomical strips parallel to the CS: anteprecentral, precentral (M1) non‐proper, precentral (M1) proper, and postcentral. The subdivision into M1 proper and M1 non‐proper was determined according to the anatomical course of the precentral gyrus and CS, reflecting the assumed underlying structural organization of the cortex. Ten stimulation sites were targeted in each strip, applying a stimulation intensity of 150% RMT. Consecutively, the mean electric field strength during stimulation was 94 V/m. Stimulation was initiated at the basal (temporal) starting point and sequentially applied along the predefined lines, beginning with M1 non‐proper, followed by M1 proper, then the anteprecentral, and finally the postcentral line. EMG amplitudes and latencies were analyzed post hoc.

### 2.3 Data Processing and Statistical Analysis

Data processing was conducted using RStudio 2024.09.1. Continuous data are reported as mean ± standard deviation. Distributional assumptions were explored using visual inspection of histograms and Q–Q plots. Generalized linear mixed‐effects models were applied to evaluate differences between stimulation sites. For dichotomous motor response data (presence vs. absence of a valid MEP), a binomial distribution with a logit link function was used. Continuous amplitude and latency data were analyzed using linear mixed‐effects models assuming a Gaussian distribution with an identity link. Subject was included as a random intercept in all mixed‐effects models. No formal correction for multiple comparisons was applied, as the analyses were hypothesis driven and focused on predefined contrasts between stimulation strips. Given the exploratory nature of the study and the limited sample size, results were interpreted conservatively with an emphasis on effect estimates rather than isolated *p*‐values. A *p*‐value of < 0.05 was considered statistically significant. Graphical representations were generated using GraphPad Prism 10.

## 3 Results

### 3.1 Variation of Amplitudes With Stimulation Area

A generalized linear mixed model with a binomial distribution and logit link was applied to the dichotomous motor response data. Stimulation site was included as a fixed effect. The analysis was based on 600 stimulation trials. The fixed effect of stimulation site was not statistically significant (*F* = 0.869, *p* = 0.457). Descriptively, valid motor responses occurred in 106 of 150 stimulations at the M1 non‐proper site (70.7%), 110 of 150 at the M1 proper site (73.3%), 99 of 150 at the anterior precentral site (66.0%), and 110 of 150 at the postcentral site (73.3%).

**FIGURE 2:**
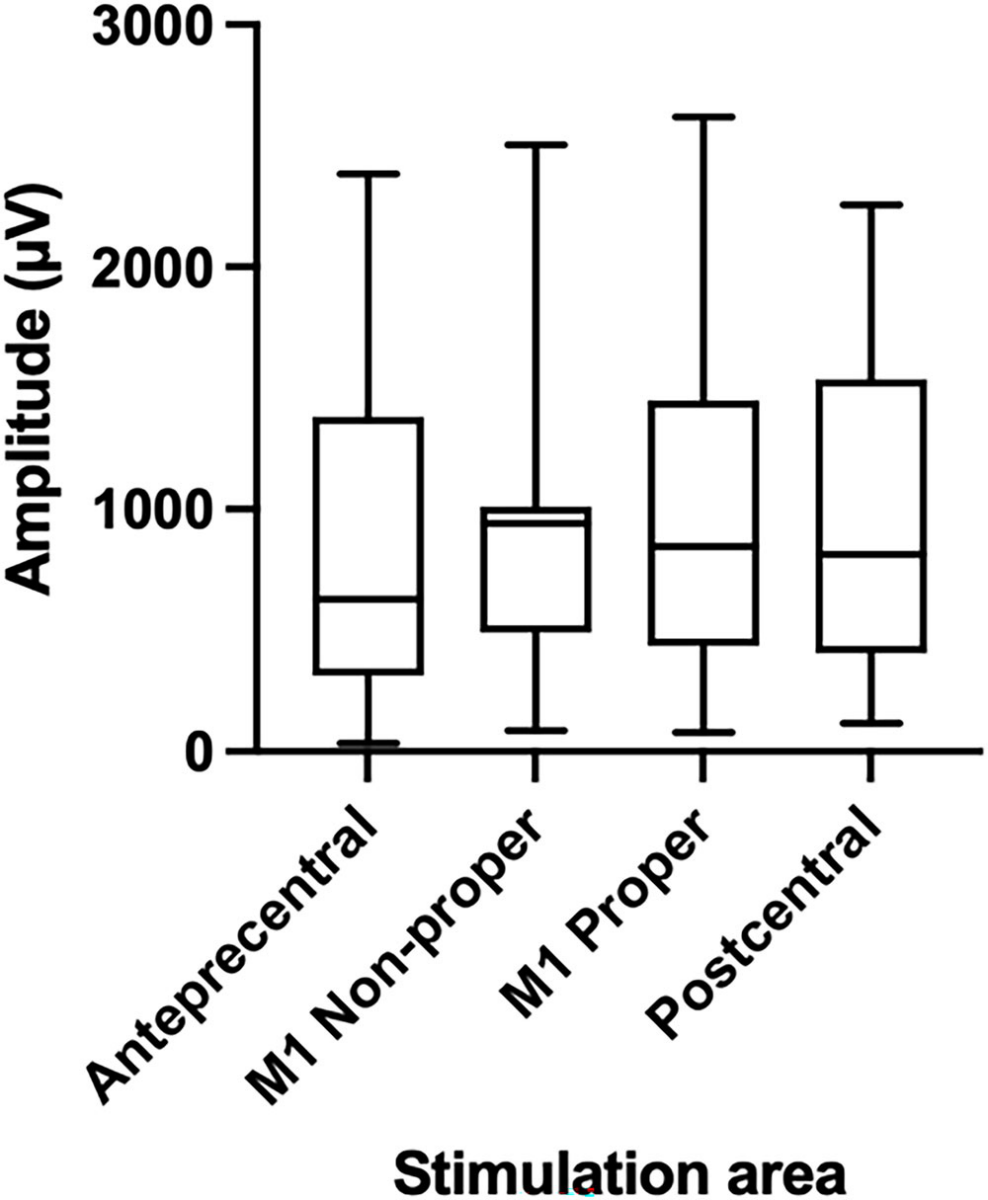
Boxplots of mean motor‐evoked potential (MEP) amplitudes across the four stimulation sites (M1 non‐proper, M1 proper, anterior precentral, and postcentral). No significant differences in mean MEP amplitude were observed between stimulation sites (linear mixed‐effects model, *p* = 0.794).

A linear mixed‐effects model was then fitted to the amplitude data with stimulation site as a fixed effect and subject as a random intercept. This analysis also revealed no significant main effect of stimulation site on MEP amplitude (*F* = 0.343, *p* = 0.794), indicating that mean amplitudes did not differ significantly between the investigated regions. The results are summarized in Table 1 and illustrated in Figure 2.

**TABLE 1:** Motor response characteristics across stimulation sites. The table summarizes response rates, mean motor evoked potential (MEP) amplitudes, and latencies for the four anatomical stimulation sites (M1 non‐proper, M1 proper, anterior precentral, and postcentral), based on mixed‐effects model analyses showing no significant main effect of stimulation site.

Subject	Anteprecentral					M1 non‐proper					M1 proper					Postcentral				
	Amplitude (µV)			Latency (ms)	V/m	Amplitude (µV)			Latency (ms)	V/m	Amplitude (µV)			Latency (ms)	V/m	Amplitude (µV)			Latency (ms)	V/m
	Mean	Min	Max	Mean	Mean	Mean	Min	Max	Mean	Mean	Mean	Min	Max	Mean	Mean	Mean	Min	Max	Mean	Mean
1	374	81	1322	25.3	140.7	1383	77	3837	20.7	142.6	2436	74	6330	21.4	141.7	1016	91	2202	18.9	141.1
2	761	80	1983	22.9	101.0	669	183	1301	23.3	92.0	673	36	1860	23.8	94.1	814	336	1415	22.2	69.7
3	2364	74	7308	19.7	96.6	1220	224	2842	18.0	87.8	1286	172	2127	22.8	89.6	2311	527	4531	19.9	91.2
4	158	72	291	19.4	114.5	109	51	181	20.6	113.2	112	53	193	20.1	110.3	164	62	269	18.2	112.1
5	2304	176	4222	21.3	107.5	2689	73	4895	20.5	113.2	2493	48	7346	21.6	112.8	2919	30	4660	19.7	116.3
6	1319	76	2188	23.9	95.4	793	138	2009	22.8	92.9	302	102	520	23.0	88.0	451	79	1098	23.6	89.5
7	4765	609	8396	19.9	63.6	4425	588	7522	19.6	60.5	3742	746	6997	20.1	59.9	1920	69	6670	20.6	58.8
8	1251	34	4427	21.7	70.3	750	16	1123	21.9	73.3	1203	44	3439	19.5	73.7	350	73	1246	21.4	72.2
9	579	228	1122	22.8	121.5	1624	30	2363	24.2	122.4	510	33	1043	23.3	112.9	946	109	2203	22.9	116.1
10	1897	498	5664	21.0	85.1	1299	678	1819	21.9	87.9	2414	302	5006	20.6	84.4	1859	682	3058	21.8	83.4
11	398	50	1078	27.1	80.5	1082	72	3159	19.3	79.7	733	53	1721	20.1	78.2	698	77	1947	24.7	77.9
12	532	168	860	18.8	70.8	1181	416	2505	19.7	70.7	776	93	1659	19.9	70.5	758	37	1840	20.9	69.2
13	3904	1620	6776	18.9	107.3	1564	37	4532	19.8	108.4	1947	59	4216	19.8	105.0	1521	349	3265	18.7	102.3
14	858	38	2295	22.6	150.6	1130	200	2101	20.9	147.5	726	326	1175	22.7	148.0	1374	37	3029	22.5	146.1
15	1764	51	3717	21.6	78.7	3576	88	5859	20.9	80.8	1663	15	3173	25.7	80.1	3758	388	6954	21.4	77.2
Column mean	1548	257	3443	21.8	98.9	1566	191	3070	20.9	98.2	1401	144	3120	21.6	96.6	1391	196	2959	21.2	94.9

### 3.2 Variation of Latencies With Stimulation Area

**FIGURE 3:**
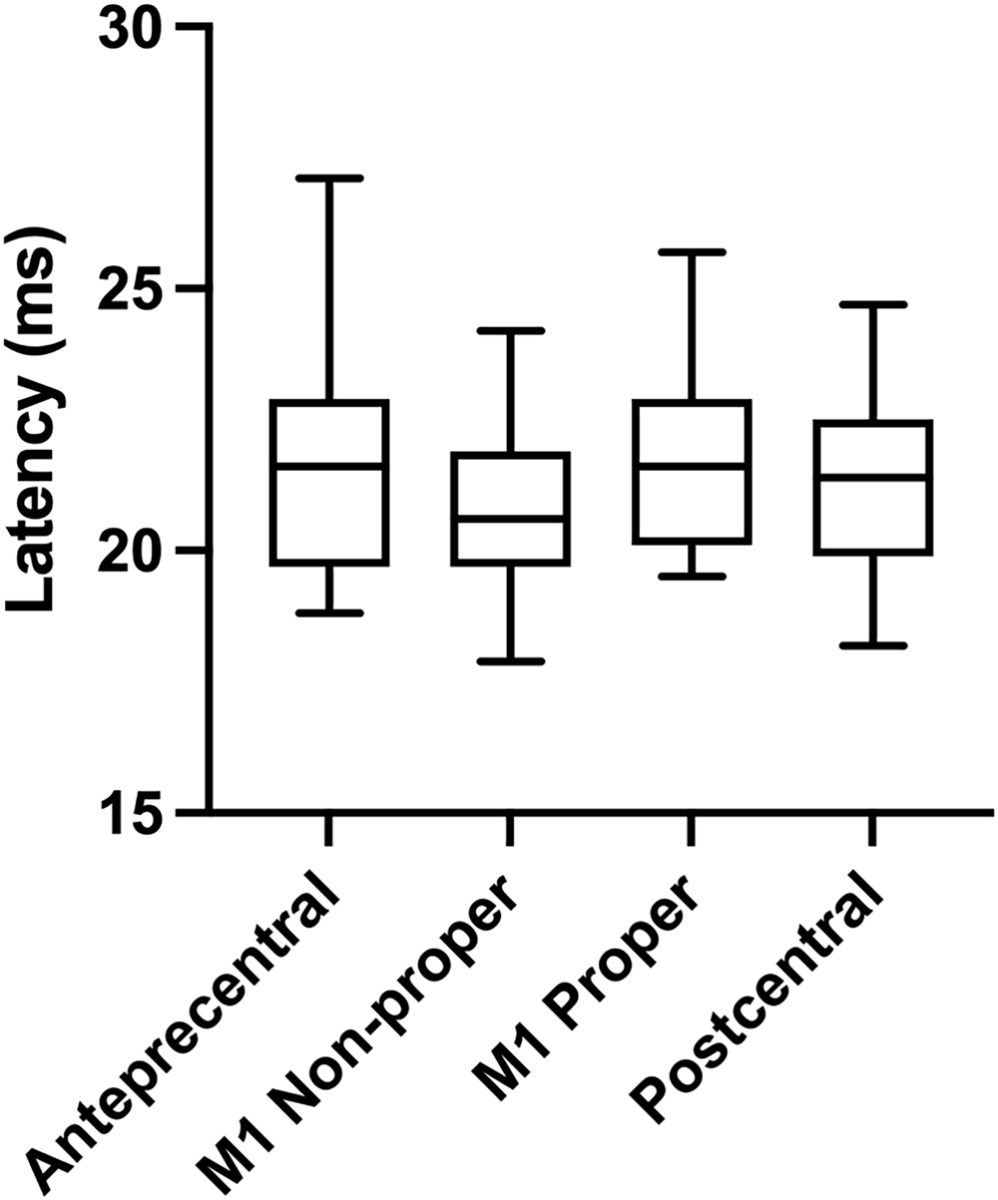
Boxplots of motor‐evoked potential (MEP) latencies across the four stimulation sites (M1 non‐proper, M1 proper, anterior precentral, and postcentral). No significant differences in MEP latency were observed between stimulation sites (linear mixed‐effects model, *p* = 0.528).

A linear mixed‐effects model was fitted to the latency data with stimulation site as a fixed effect and subject as a random intercept. The analysis revealed no significant main effect of stimulation site on MEP latency (*F* = 0.741, *p* = 0.528), indicating that response latencies were comparable across the four anatomical regions. Mean latencies were 21.0 ± 0.6 ms for the M1 non‐proper site, 21.7 ± 0.6 ms for the M1 proper site, 22.0 ± 0.6 ms for the anterior precentral site, and 21.2 ± 0.5 ms for the postcentral site. These findings are presented in Table 1 and visualized in Figure 3.

**FIGURE 4:**
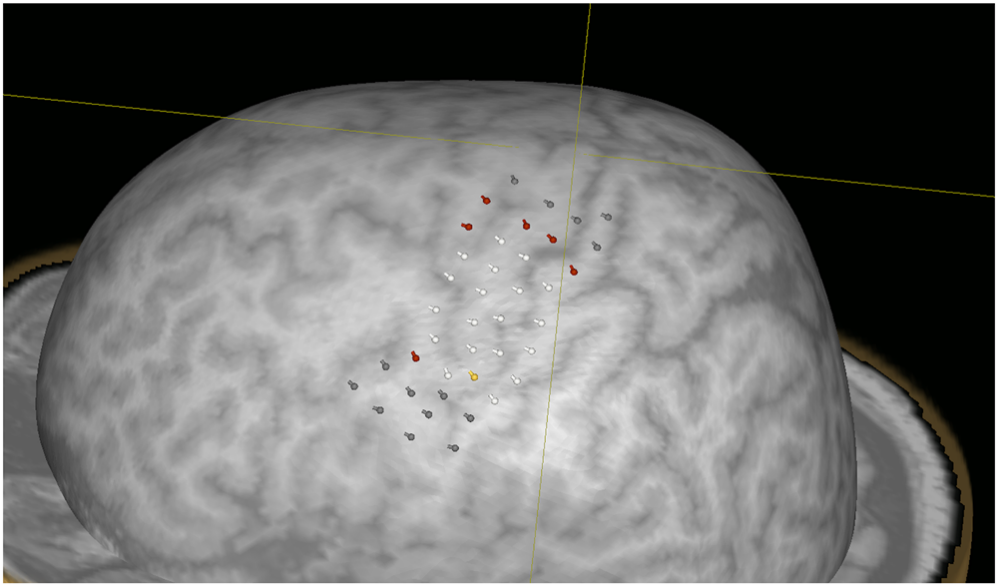
Exemplary case of the performed mapping protocol on the left hemisphere.

In Figure 4, an exemplary case of the performed mapping protocol is portrayed.

## 4 Discussion

NTMS enables functional mapping with a high degree of spatial accuracy. Since the early days of TMS navigation, the precision of nTMS mapping has been of interest for researchers (Boroojerdi et al. 1999). With the advent of increasingly precise navigation systems, various dimensions of accuracy have been explored, including interobserver and intraobserver reliability (Sollmann et al. 2013; Wolf et al. 2004). Furthermore, comparative studies investigating the concordance between the gold standard of motor mapping—direct cortical stimulation (DCS)—and nTMS have demonstrated a reliable spatial correlation (Lucas et al. 2020; Picht et al. 2011). In these studies, Picht et al. found a mean discrepancy of only 4–7 mm between DCS and nTMS motor hotspots, while Lucas et al. observed an average discrepancy of 11 mm. These findings support the feasibility of replicating Rossi's anatomical subdivisions of the primary motor cortex using a non‐invasive nTMS approach.

By translating Rossi et al.’s findings into an nTMS model, we aimed to investigate potential differences in MEP amplitudes and latencies. While we hypothesized that the faster motor response associated with the M1 proper region would be reflected in shorter TMS‐induced latencies, our results did not reveal a statistically significant difference between suspected proper and non‐proper areas. Further, we interpreted the higher excitability of the M1 proper region as the capacity to elicit greater EMG response amplitudes. Although our data showed a trend toward higher MEP amplitudes in the M1 proper region, this difference did not reach statistical significance.

To account for potential navigation inaccuracies—including the reported TMS deviation of 4–11 mm as described by Picht et al. and Lucas et al.—we included additional mapping rows anterior (anteprecentral) and posterior (postcentral) to the primary region of interest. However, comparative analyses across all four mapped rows did not reveal any significant differences in the measured parameters. Consequently, it appears unlikely that navigation inaccuracy accounts for the lack of observed correlation in our findings.

Another critical element of cortical mapping, the stimulation frequency, poses challenges in translating into a TMS model. Standard DCS mapping utilizes both low‐frequency (LF) and high‐frequency (HF) protocols. In the cited study, an HF train‐of‐5 (To5) protocol is predominantly used for motor mapping. In order to identify M1 properly, however, the stimulation frequency is subsequently adjusted to a train‐of‐two (To2) protocol. For classical TMS motor mapping, a single‐pulse stimulation is used. A change in the frequency of pulse repetition leads to repetitive, inhibitory pulses (starting at a frequency of 1 Hz) and thus cannot be considered a substrate for frequency modulation in DCS. However, the paired‐pulse technique (which our device unfortunately does not support) may approximate this variable more closely. In a recently published study, Madsen et al. investigated exactly this difference in TMS stimulation of the hand area and found that paired‐pulse stimulation results in a more dorsally located center of gravity (COG) of stimulation compared to conventional single‐pulse stimulation (Madsen et al. 2025). This aligns well with our above‐mentioned hypothesis and suggests that a distinction between M1 proper and non‐proper may be possible with paired pulse protocols.

Another point worth considering is the fact that “higher excitability” could alternatively be interpreted as “more easily excitable,” which may have warranted a different experimental setup. Rather than focusing on reliably eliciting responses with an RMT of 150%, a stimulation protocol closer to the motor threshold (RMT 100%) might have allowed for a more sensitive differentiation between neuronal motor networks based on their excitability threshold.

Another influencing factor on TMS stimulation is coil orientation. According to the existing literature, optimal motor responses in the hand area are achieved when the induced current is oriented perpendicular to the targeted gyrus (Gomez‐Tames et al. 2018; Siebner et al. 2022). Although it cannot be excluded that varying coil orientation angles might allow for further differentiation between cortical subregions, this aspect was not addressed in the present study.

On a cytoarchitectonic level, the motor cortex is classically divided into six layers (lamina I molecularis, II granularis externa, III pyramidalis externa, IV granularis interna, V pyramidalis interna, and VI multiformis). Layer V of the motor cortex is typically known for containing giant pyramidal cells, named after their discoverer Wladimir Betz (Betz 1874). Experimental TMS studies have shown that it is primarily these layer V cells that are activated by TMS, with a lesser extent of activation also observed in cells of layers II and III (Aberra et al. 2020). In their multimodal 3D atlas of the macaque motor cortex, Rapan et al. (2021)  were able to demonstrate significant differences in the cytoarchitecture of the anterior and posterior part of the motor cortex (area 4a and 4p in their nomenclature). While area 4a shows a lower cell‐body packing density in layers I, III, and VI, 4p shows an additional enlargement of layers II and Vb (due to the presence of Betz cells). These differences in cell composition make a differentiation by means of TMS appear plausible.

Furthermore, the terminology applied in the present study (and in Rossi et al.) to describe the broader motor region warrants further discussion. Depending on the author, the term M1 is used to refer to a morphological description of the precentral gyrus. Within this region, a functional subdivision—termed M1 proper and non‐proper M1 in this case—can be identified by DCS. In the original descriptions by Penfield and Brodmann, however, the terminology was primarily based on different cell clusters, reflecting stimulation and functional properties rather than surface anatomy. The term M1 (Penfield) and BA4 (Brodmann) was classically used to describe the cytoarchitecture corresponding to what we refer to here as M1 proper, located in the dorsal portion of the precentral gyrus. Accordingly, the search within the precentral gyrus can also be interpreted as a distinction between Brodmann areas BA4 and BA6. BA4 contains the aforementioned Betz cells (giant pyramidal neurons) in layer V.

TMS has been successfully used in prior studies to differentiate between the primary motor cortex and the premotor cortex (Bäumer et al. 2003; Fiori et al. 2018; Fleischmann et al. 2021; Lega et al. 2020; Vesia et al. 2018). However, the examined area was located more caudally compared to our examination in the ventral premotor cortex (PMv) in Fiori's case or based on a virtual lesion protocol in Fleischmann's examination.

A comparable differentiation with the presented motor TMS single pulse protocol was not feasible.

### 4.1 Limitations

Several limitations of the present study must be acknowledged. First, the relatively small sample size (*n* = 15) limits statistical power, particularly for detecting subtle regional differences in EMG amplitude and latency. While the total number of stimulation trials was high, inter‐individual variability in cortical excitability may not be fully compensated by repeated measures. Future studies with larger cohorts or Bayesian modeling approaches may be better suited to quantify evidence for or against small effect sizes.

### 4.2 Conclusion

While nTMS demonstrated high accuracy in the localization of motor function, the applied protocol did not permit a clear function delineation between a ventral and dorsal subregion within the precentral gyrus. It is worth noting that the use of relatively high stimulation intensities may have obscured subtle differences that could potentially be detected under different conditions. Future studies employing lower stimulation intensities—such as stimulation at 100% of the RMT—or a specific paired pulse protocol may provide improved sensitivity for distinguishing between these anatomically and functionally distinct areas.

## Author Contributions

Conceptualization and methodology: G.D. Software: R.B. Validation: G.D., A.P., and R.B. Formal analysis: G.D., R.B., and G.C. Investigation: G.D., G.C., G.S., V.M., L.S., and H.G.K. Resources: G.D. and A.P. Data curation: G.C. and R.B. Writing – original draft preparation: G.D. and R.B. Writing – review and editing: G.D., A.P., R.B., and N.G. Supervision: A.P. and C.R.W. Project administration: G.D. All authors have read and agreed to the published version of the manuscript.

## Funding

The authors have nothing to report.

## Ethics Statement

The study was approved by our local ethics committee (Ulm University, Nr. 333/24).

## Consent

No patients were examined in this study. All healthy participants gave informed consent.

## Conflicts of Interest

The authors declare no conflicts of interest.

## Data Availability

The data that support the findings of this study are available on request from the corresponding author. The data are not publicly available as they are containing information that could compromise the privacy of research participants.
